# Design, Manufacture, and Characterization of a Critical-Sized Gradient Porosity Dual-Material Tibial Defect Scaffold

**DOI:** 10.3390/bioengineering11040308

**Published:** 2024-03-25

**Authors:** Ming-Chan Lee, Cheng-Tang Pan, Wen-Fan Chen, Meng-Chi Lin, Yow-Ling Shiue

**Affiliations:** 1 Department of Electrical Engineering, National Kaohsiung University of Science and Technology, Kaohsiung 807, Taiwan; mclee@nkust.edu.tw; 2 Institute of Advanced Semiconductor Packaging and Testing, College of Semiconductor and Advanced Technology Research, National Sun Yat-Sen University, Kaohsiung 804, Taiwan; pan@mem.nsysu.edu.tw; 3 Department of Mechanical and Electro-Mechanical Engineering, National Sun Yat-Sen University, Kaohsiung 804, Taiwan; 4 Institute of Precision Medicine, National Sun Yat-Sen University, Kaohsiung 804, Taiwan; 5 Taiwan Instrument Research Institute, National Applied Research Laboratories, Hsinchu City 300, Taiwan; 6 Institute of Medical Science and Technology, National Sun Yat-Sen University, Kaohsiung 804, Taiwan; sallychen@imst.nsysu.edu.tw; 7 Department of Surgery, Zuoying Armed Forces General Hospital, Kaohsiung 813, Taiwan; 8 Institute of Biomedical Sciences, National Sun Yat-Sen University, Kaohsiung 804, Taiwan

**Keywords:** finite element analysis, radial gradient porosity, stress concentration elimination, Ti64 ELI, selective laser melting, uniform design method, nanoindentation, strain energy

## Abstract

This study proposed a composite tibia defect scaffold with radial gradient porosity, utilizing finite element analysis to assess stress in the tibial region with significant critical-sized defects. Simulations for scaffolds with different porosities were conducted, designing an optimal tibia defect scaffold with radial gradient porosity for repairing and replacing critical bone defects. Radial gradient porosity scaffolds resulted in a more uniform stress distribution, reducing titanium alloy stiffness and alleviating stress shielding effects. The scaffold was manufactured using selective laser melting (SLM) technology with stress relief annealing to simplify porous structure fabrication. The study used New Zealand white rabbits’ tibia defect sites as simulation parameters, reconstructing the 3D model and implanting the composite scaffold. Finite element analysis in ANSYS-Workbench simulated forces under high-activity conditions, analyzing stress distribution and strain. In the simulation, the titanium alloy scaffold bore a maximum stress of 122.8626 MPa, while the centrally encapsulated HAp material delivered 27.92 MPa. The design demonstrated superior structural strength, thereby reducing stress concentration. The scaffold was manufactured using SLM, and the uniform design method was used to determine a collection of optimum annealing parameters. Nanoindentation and compression tests were used to determine the influence of annealing on the elastic modulus, hardness, and strain energy of the scaffold.

## 1. Introduction

There has been considerable attention and discourse in clinical medicine, especially regarding the repair of critical-sized bone defects. This is particularly pertinent to invasive implant biocompatibility and wound-healing outcomes post-surgery. Historical constraints in materials and processing techniques have traditionally led to the use of biocompatible medical-grade titanium alloys and straightforward porous structure designs when addressing extensive bone defects. However, the substantial rigidity disparity between titanium alloys and bone tissue and the non-degradable nature of these materials hampers the short-term regeneration of the surrounding bone [1,2]. This limitation results in stress concentration at the interface between implants and bone, inducing stress shielding effects. Consequently, the regenerative efficacy of bone tissue is compromised, posing significant risks such as implant migration and collapse during the recovery period [3]. This study addresses this concern by incorporating a gradient porosity bone scaffold. By varying the porosity at different levels, we aim to eliminate stress concentration points while ensuring that the mechanical strength of other parts remains relatively unchanged.

Bone transplantation surgery involves surgically replacing existing bone tissue with substitute materials. This procedure can be categorized into autografts, allografts, and synthetic bone grafts based on the type of substitute employed. Autografts utilize bone fragments obtained from other parts of the patient’s body, while allografts involve using bone from donors of the same type (allogeneic bone). Synthetic bone grafts are crafted from various materials. Artificial bone replacement materials commonly include bioceramics, metal alloys, and composite materials [4,5]. Historically, traditional medical techniques predominantly relied on autografts and allografts. Despite exhibiting superior bone formation rates and regenerated bone volume during bone growth [6], the search for suitable substitutes persists. Moreover, autograft and allograft bone transplants carry inherent risks of complications, such as rejection and infection [7]. Allografts require the careful consideration of factors like potential virus infections from the transplant source, the presence of immune disorders, and the impact of human leukocyte antigen compatibility [8,9]. In recent years, artificial bone transplantation surgeries with a stable supply of replacement sites and sources have been employed for treatment, significantly reducing patient wait times for replacement sites.

In past structural design studies, researchers drew inspiration from the architecture of bone tissue by incorporating porous structures into materials. Through in vivo experiments, implants made of the same material with added porous structures demonstrated advantages such as increased tissue permeability, thereby promoting a faster bone tissue growth rate [10,11]. Hulbert and their research team conducted experiments using ceramic materials, testing discs with and without pores. Their observations indicated a more rapid healing process around the porous discs made of the same material, with tissue fibers better enveloping the porous structure [12]. Naoya Taniguchi et al. proposed the fabrication of porous implants using titanium alloy material and observed favorable bone tissue growth in pores ranging from 300 to 600 μm in diameter [13]. Furthermore, the titanium scaffold with a 50% porosity exhibited lower maximum von Mises stress [14], while the scaffold with a 70% porosity demonstrated optimal cell proliferation and bone ingrowth capacity [15].

Selecting substitute materials is crucial for the success of artificial bone transplantation procedures. Presently, the prevalent materials utilized in medical substitutes comprise bioceramics and metal alloys. Bioceramics, represented by hydroxyapatite (HAp), are esteemed for their robust biocompatibility, non-toxicity, and osteoconductive properties. HAp is notable for its ability to control degradation in the presence of biological entities [16], contributing to its widespread utilization in artificial bone replacements. Its exceptional biocompatibility significantly accelerates the bone-healing process. However, findings from tensile testing conducted by Kutz et al. [17] reported a yield strength of 0.043 GPa, limiting the application of HAp to low-load-bearing scenarios. Experimental studies by Arcos et al. [18] revealed that a synergistic combination of HAp and metal in bone defect replacement notably enhances tissue growth efficiency. Furthermore, the results of studies by Shah et al. [19] underscored the remarkable adaptability of titanium structures to natural bone tissue. Titanium alloys, depending on the composition of different metallic elements and their lattice arrangements at room temperature, can be classified into three main categories: (1) α (hexagonal close-packed) phase alloys, (2) β (body-centered cubic) phase alloys, and (3) α + β phase alloys [20]. This study uses the third type of Ti64 ELI. It combines the high-temperature creep and oxidation resistance of α phase alloys with the high strength and flexibility of β phase alloys [21]. Conforming to the international to ASTM F136 material specifications for use in medical implants, the main components of the Ti64 ELI material, expressed in weight percentage (wt.%), include approximately 88.9% titanium, 6.5% aluminum, and 4.5% vanadium. The low-modulus alloy of Ti has also been employed in scaffold research. Luo et al. investigated the mechanical properties, biocompatibility, and proteinomics of the low-modulus alloy Ti-Nb-Ta-Zr [22]. Song et al. achieved low-modulus radial gradient gyroid porous Ta structures via laser powder bed fusion [23].

In addition to excellent biocompatibility, some alloys possess the appropriate combination of properties for use in high-load-bearing applications in the body, an example being medical-grade titanium alloy (Ti64 ELI). It boasts superior tensile strength without structural limitations (elastic modulus = 80–130 GPa [24]) and excellent corrosion resistance and wear resistance [25]. A study by Zhang et al. [26] highlighted the practical support provided by artificial bone replacements fabricated from Ti alloy during the healing process. Rigorous sterilization procedures significantly mitigate issues such as infections. However, subsequent studies found that the elastic modulus of the alloy is substantially higher than that of cortical bone (17 GPa) and bone (5 GPa) of the distal tibia in non-osteoporotic middle-aged men [27,28]. This discrepancy results in uneven force distribution post-implantation, leading to excessive stress concentration and the formation of a stress shielding effect. This phenomenon diminishes the regenerative efficacy of bone tissue and heightens the risk of implant slippage and collapse during the recovery process [29].

Li et al. introduced an innovative topology optimization methodology that concurrently considers mechanical and fluidic properties in the design of porous scaffolds [30]. Wang et al. investigated the effects of porosity, pore size, and radial pore size distribution on compressive mechanical properties, cellular responses, and bone regeneration outcomes [31]. This study introduces an optimized scaffold design approach for addressing critical-sized tibial defects. This study employs Ansys-Workbench for simulating post-implantation stress distribution and utilizes selective laser melting (SLM) to streamline the production of various structures by manufacturing physical prototypes. Subsequently, automated testing verifies the scaffold’s mechanical performance to ensure robust support provision.

The chosen manufacturing material is medical-grade Ti64 ELI alloy, integrating a gradient porous structure and bone plate into the overall formation. To promote bone tissue growth, HAp was selected for the central part of the scaffold. Utilizing Ti alloy with a gradient porous structure minimizes interface stress concentration between the scaffold and bone defect during bone growth. This design facilitates optimal bone support while safeguarding the HAp material from excessive compression and potential fractures during the initial implantation process. The scaffold is securely affixed using interconnected bone plates, aiding surgeons in achieving swift positioning during surgery and installing the scaffold at bone-cutting sites. This mitigates the risk of post-operative migration and significantly reduces the likelihood of complications. Our team has conducted previous studies on bone defects [32,33].

The current work comprises three main components. Firstly, finite element analysis (FEA) is employed to simulate the mechanical responses of scaffolds with different porosities and materials under high-activity conditions in rabbits. Secondly, a uniform design method (UDM) significantly reduces the experimental iterations required to explore the optimal annealing temperature and duration. The structures are manufactured using SLM to address various locations of bone defects, significantly reducing manufacturing time compared to traditional processes. Subsequently, annealing is performed at the optimal temperature and duration parameters to reduce stress concentration in the scaffold and achieve homogenization. Thirdly, nanoindentation tests are conducted on the prototype scaffold to obtain three mechanical properties (modulus, hardness, and strain energy) and investigate the scaffold’s structural strength and stress concentration.

## 2. Experimental Methods

This study focused on a Ti64-ELI-based porous scaffold for the critical-sized tibial defect. The work was divided into scaffold design and simulation, followed by manufacturing. CAD software (SolidWorks 2017) was used for scaffold design, and FEA was employed to model tibial stress distribution post-implantation. A prototype was manufactured using SLM and optimized stress relief annealing parameters. Structural strength was determined through mechanical performance testing.

### 2.1. Bone Model Construction

**Figure 1:**
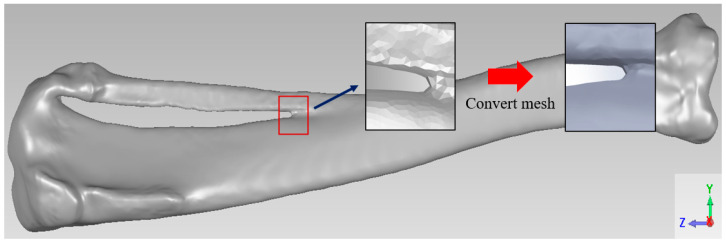
Tibia model.

This study utilized adult New Zealand White rabbits (NZW) tibiae as experimental subjects, introducing a segmental bone defect in the midshaft. Computed tomography (CT) scanning was employed to proportionally scale and reconstruct the complete skeletal structure in a 3D model. Subsequently, Meshmixer software 3.3 (Autodesk Inc., San Rafael, CA, USA) processed the original bone model, extracting the specific left tibia portion needed for the study, performing surface smoothing, and repairing model damages. Finally, Geomagic Studio software for SolidWorks 2017 (3D Systems Inc., Rock Hill, SC, USA) facilitated the parametric surface setup to create highly fitting surface patches. The resulting tibia model is depicted in Figure 1.

### 2.2. Design of Scaffold for Bone Defect

#### 2.2.1. Bone Plate and Porous Titanium Cage Integrated Forming Structure

**Figure 2:**
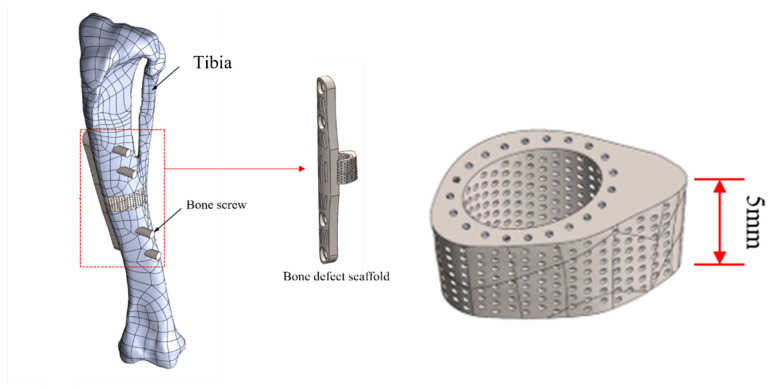
Schematic illustration of composite structural scaffold integrated with bone model.

This study introduces a novel composite scaffold aimed at enhancing stress distribution. The structure comprises two main components: a titanium alloy framework for the scaffold, forming a porous Ti alloy cage (height = 5 mm), with an external shape tailored to the curvature of the original skeletal structure; a second component, a 40 mm-long, 5 mm-wide, and 2 mm-thick curved titanium bone plate designed to accommodate the irregular bone curvature of rabbit tibiae. This plate is integrated with the titanium cage and secured using four 2 mm-diameter bone screws. The design facilitates adequate internal fixation and the prevention of extensive displacement during the recovery period, as shown in Figure 2.

#### 2.2.2. Radial Gradient Porosity Structure Design

In the internal structure design of the titanium cage, the primary goal is to facilitate the successful attachment of newly formed bone tissue through the optimal average pore size range for bone tissue regeneration, explicitly ranging between 50 and 600 μm. From a mechanical standpoint, careful considerations are made to ensure a uniform distribution of load-bearing forces post-implantation, thereby mitigating issues related to stress concentration. The design strategy involves dividing the titanium cage into five distinct layers (70%, 65%, 60%, 55%, and 50%), gradually reducing porosity from the end near the bone plate. The section near the bone plate offers enhanced support and can withstand higher loads. In contrast, the opposite end, lacking bone plate support, bears relatively lower loads, resulting in uneven distribution. Controlling radial gradient porosity, a higher porosity is designed at the end near the bone plate, gradually decreasing in each subsequent layer. This approach can effectively resolve uneven load distribution issues, ensuring the overall stability of the Ti cage and optimal conditions for post-implantation regeneration within its structure.

### 2.3. Finite Element Analysis

#### 2.3.1. The Mixing Rules for Metal Composite Materials

**Figure 3:**
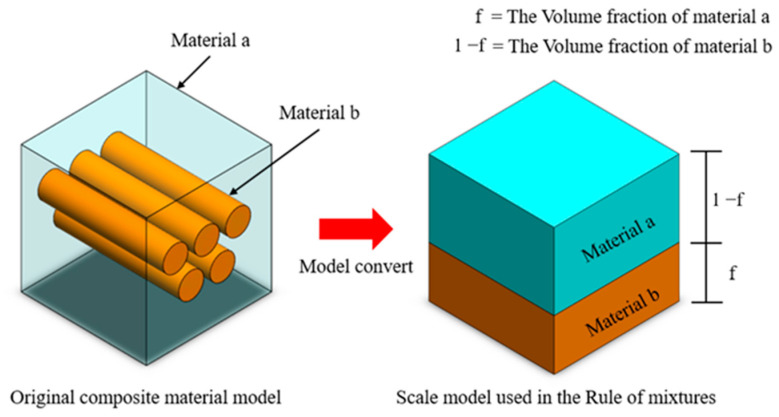
The rule of mixture for the stratified model of composite material volume fraction.

**Figure 4:**
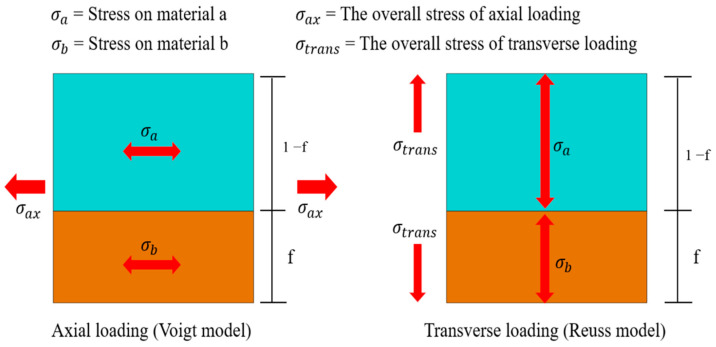
The rule of mixture for the two-way loaded free body diagram.

In materials science, the mixture rule is a concise and precise estimation method used primarily for predicting the mechanical properties of metal composites with diverse elemental compositions. These properties encompass parameters such as Young’s modulus, mass density, and Poisson’s ratio. The approach relies on the distinctions in volume ratios of constituent elements within varying composite materials. Initially formulated as a straightforward proportional layer model, as depicted in Figure 3, subsequent steps involve establishing Voigt and Reuss models based on the principles of equivalent strain and equivalent stress in materials mechanics, as illustrated in Figure 4. Ultimately, employing a weighted averaging method leads to the derivation of two sets of conversion formulas, facilitating the computation of upper and lower bounds for the material’s mechanical properties.

#### 2.3.2. Boundary Condition Settings

This study utilized Ansys-Workbench for finite element analysis, simulating the implantation of a radial gradient porosity scaffold into the rabbit tibia’s defect area. It evaluated the impact of diverse internal porous structures and material compositions on bone tissue’s load-bearing performance. Six sets of mechanical finite element analyses were conducted, as shown in Table 1. Initially, a 5 mm bone block autograft was a standard control for comparing actual bone tissue replacement with the designed artificial bone. Subsequently, the titanium alloy scaffold’s internal structure was modified into three groups: 50% single porosity, 70% single porosity, and radial gradient porosity, confirming the stress distribution under different structural conditions. Finally, two groups were compared regarding material composition differences: one lacked space for placing bioceramics in the porous titanium alloy scaffold, and the other included HAp in the space in the porous Ti alloy scaffold.

In simulating force boundary conditions, we considered the force magnitude on the rabbit tibia during high activity, focusing on the force range at the tibial joint connection. This force magnitude design is based on the findings of Gushue et al. [34], where the upper femoral contact surface is categorized into inner and outer sides. The force direction is determined with insights from the study by Perez et al. [35], considering the mechanical impact of muscles and ligamentous tissues. The main force is the axial Fz, which is 77.1 N inside and 89.3 N outside. The lateral forces Fx and Fy are 0 N, and the lower tibia is fixed at two points.

### 2.4. Ti64 ELI Powder Fusion Processing

#### 2.4.1. Ti64 ELI Powder Fusion

This study employed the fully automated SLM machine EOSINT M280 for sample fabrication. The equipment utilizes layer-by-layer manufacturing technology, eliminating the need for traditional machining tools, significantly reducing processing time, and offering high flexibility for customized part production in a short timeframe. Ti64 ELI powder, designed explicitly for EOSINT M systems and provided by the German company EOS, was used in manufacturing. The machine features a sample building space of 250 mm × 250 mm × 325 mm, a maximum laser power of 200 W, a scanning speed of up to 7000 mm/s, a scanning beam diameter ranging from 100 to 500 μm, and a single-layer processing thickness of 20–40 μm. These specifications meet the spatial and porosity formation precision requirements for the sample fabrication in this study.

#### 2.4.2. High-Temperature Stress Relief Annealing Process

Annealing was carried out in air. The annealing parameters used included heating rate, duration, and cooling method. Based on these conditions, a UDE with six levels and four factors was used (Table 2). The mark “*” means it has better uniformity than other UDE tables. Table 3 shows the use form for choosing the type of U^*^_6_ (6^4^) table, S is factor number, and D means discrepancy.

### 2.5. The Forming Results and Mechanical Property Measurements

#### Nanoindentation Technology

This study utilized nanoindentation to determine the mechanical property variations of the annealed scaffold, including elastic modulus and hardness. Measurements were performed with the Nano-indenter XP (MTS Systems Corporation, Eden Prairie, MN, USA), employing a Berkovich diamond tip with a 65.3-degree half angle. The triangular geometry of this tip is well-suited for high-strength metallic material measurements.

**Figure 5:**
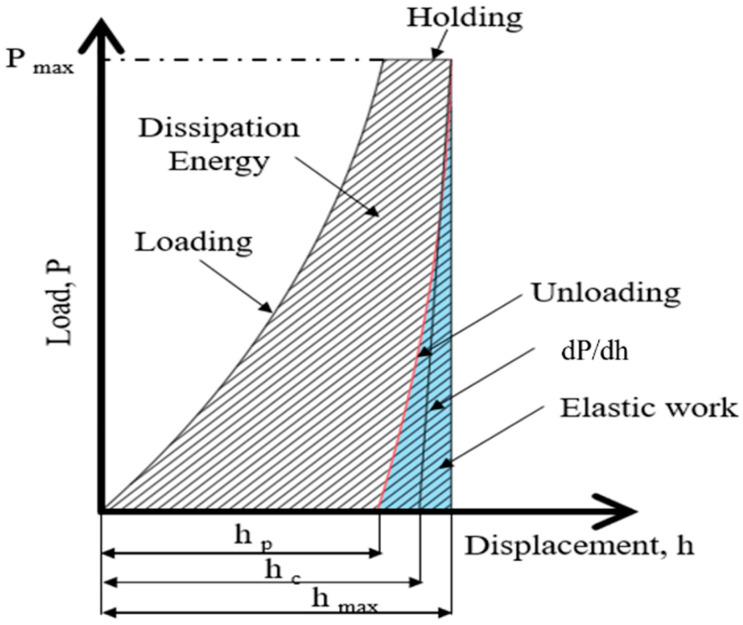
Schematic load (P)-versus-displacement (h) graph.

**Figure 6:**
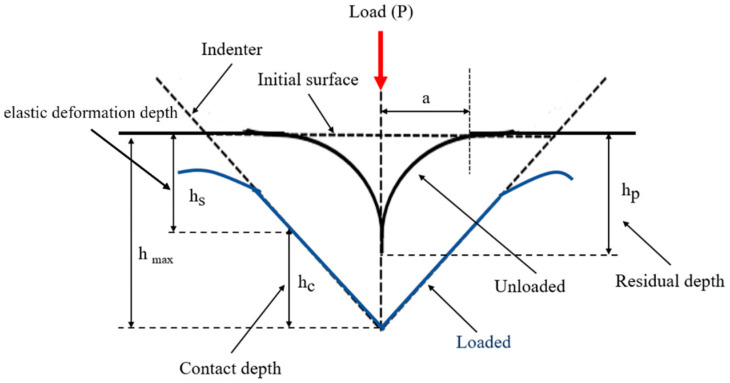
Material indentation depth profile.

Based on the principles of nanoindentation, the indenter tip makes contact with the measurement surface and progressively applies load until reaching a specific depth or load threshold, followed by unloading. This process is continuous. As the probe penetrates the sample, it induces permanent deformation on the surface. Upon unloading, the probe retracts, leaving an incompletely recovered indentation. Subsequently, the load-versus-displacement curve is generated, and the material’s indentation depth profiles are compared, as depicted in Figure 5 and Figure 6. Analyzing this curve using appropriate formulas enables the determination of the material’s Young’s modulus and hardness. From this curve, the elastic modulus and hardness of the scaffold is obtained. This study employed the Continuous Stiffness Measurement (CSM) mode integrated into the system. Arrays of nine indents were made on the embedded and polished sample surfaces before and after annealing. The spacing between each point was set at 5000 nm, with an indent depth of 2000 nm. The frequency of indentation was 45 Hz, and the rate was 10 nm/s, with specific parameters presented in Table 4.

- Compression Test

Compressive strength, a crucial metric for assessing fundamental mechanical properties and structural design strength, was investigated using a universal testing machine in this study. The experimental setup involved applying a compressive force to the test specimen through a compression plate controlled by a load motor. Deformation of the specimen under these conditions was measured using sensors. The AGS-X electronic universal testing machine (SHIMADZU, Japan) was employed explicitly for the experiments.

Mechanical simulations were performed on the samples in this study, including structural validation and compressive strength tests, to ensure the structural integrity of the implant within the rabbit tibia, mitigating potential deformation due to body weight and movement. The objective was to examine the displacement and strain induced by the bracket under controlled loading conditions. The boundary conditions for this experiment are detailed in Table 5. Three samples, both untreated and subjected to stress relief annealing, were selected, with the maximum load set at three times the body weight of an adult rabbit, approximately 150 N. The experiment involved continuous loading from 0 to 150 N, with a load rate of 0.001 kN/s. Machine-generated data provided insights into the relationship between applied load and specimen displacement during the compression test. Then, these data were converted into stress and strain units based on the sample’s contact cross-sectional area, yielding the stress-versus-strain curve, from which the compressive strength of the implant was determined. Finally, by calculating the area under the stress–strain curve, the absorbed strain energy of the specimen was determined. A comparative analysis of strain energy before and after annealing was performed to investigate stress concentration.

## 3. Results and Discussion

### 3.1. Finite Element Analysis

This study used the left tibia of New Zealand white rabbits to create a 3D model with a 5 mm critical defect. Subsequently, a porous gradient composite regenerative scaffold was designed based on this defect. Finite element analysis simulated and validated the mechanical performance of this design in an implantation environment. It was then compared with autogenous bone grafts, single-porosity titanium alloy, and gradient porous titanium alloy scaffolds in the same environment. From a mechanical standpoint, the study aimed to demonstrate two crucial design advantages: maintaining relatively stable strength and biocompatibility in the implantation environment.

#### 3.1.1. Autologous Bone Graft Block

**Figure 7:**
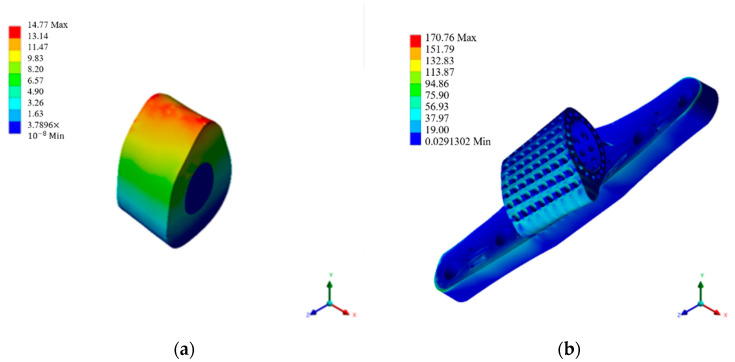

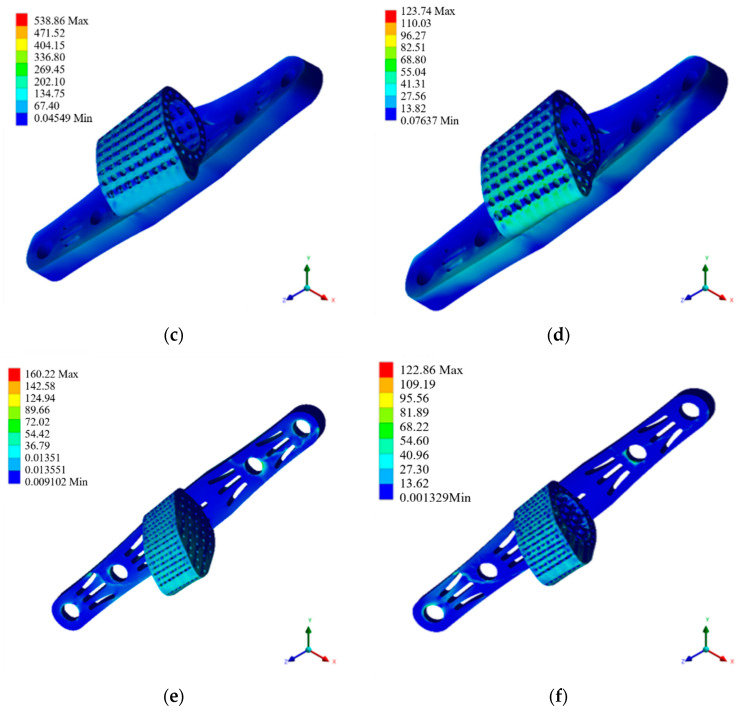
Mechanical simulation analysis diagram. (**a**) Autologous bone graft block. (**b**) 50% porosity. (**c**) 70% porosity. (**d**) Radial gradient porosity. (**e**) Ti64 ELI support body. (**f**) HAp gradient pore scaffolds.

Creating a self-bone block model matching the defect range, possessing material properties equivalent to the original skeleton, and simulating its stress under high-activity conditions, the outcomes are depicted in Figure 7a and summarized in Table 6. Subject to specified boundary loading conditions, the self-bone block model withstands a maximum stress of 14.762 MPa and experiences a maximum strain of 0.2508. Drawing on prior research by scholars highlighting the outstanding mechanical compatibility of self-bone blocks post-implantation [6], this study employed these analytical findings to guide scaffold design, aiming to closely emulate such favorable mechanical performance.

#### 3.1.2. Scaffolds with 50% and 70% Single-Porosity Made from Ti64 ELI Material

This study utilized Ti64 ELI material in the titanium alloy scaffold, employing a radial gradient porosity ranging from 50% to 70% to mitigate stress concentration issues at the scaffold interface and bone tissue interface. Through the design of a single pore connecting 50% and 70% titanium cages with the bone plate, a composite scaffold model was created, integrated with the defective tibia, and the results were cross-referenced with mechanical simulation outcomes.

The scaffold with 50% porosity, as shown in Figure 7b and Table 6, exhibits an overall model that experiences a maximum stress of 170.368 MPa and a maximum strain of 0.1584. Meanwhile, the simulation results for the scaffold with 70% porosity are presented in Figure 7c and Table 6, revealing that the overall model withstands a maximum stress of 537.636 MPa and a maximum strain of 0.4972. Comparing the simulation outcomes of the single-porosity scaffolds with varying porosity rates underscores the pronounced impact of porosity on mechanical distribution. Despite the favorable stress distribution performance of the 50% porosity titanium cage scaffold, it still falls short of the overall tibia, displaying noticeable disparities from the original bone tissue.

#### 3.1.3. Radial Gradient Porosity Scaffold with Ti64 ELI Material

The simulation of radial gradient porosity scaffolds reveals a pronounced impact of porosity control on stress distribution. This study emphasizes simulating titanium cage scaffolds with a radial gradient porosity structure, as shown in Figure 7d and Table 6. The maximum stress observed is 123.464 MPa, with a maximum strain of 0.1144. The simulated data indicate that the radial gradient porosity structure in this composite design demonstrates mechanical performance closely resembling natural bone. Consequently, this scaffold design will undergo further investigation, incorporating additional coating materials to explore any distinctions in the results.

#### 3.1.4. The Composite Scaffold Incorporating an All-Titanium Alloy Structure

The material at the center of the scaffold is divided into two types for comparison. One utilizes Ti64 ELI material, similar to the scaffold. This section discusses the simulation results and differences, as depicted in Figure 7e and Table 6. The model exhibits maximum stress and strain values of 159.852 MPa and 0.1672, respectively. Adding a titanium alloy filling to the retained cavity at the center of the scaffold, while not showing significant changes in the scaffold’s structure, demonstrates a tendency to reduce stress concentration on the bone contact surface.

#### 3.1.5. The Composite Scaffold Combined with Ti64 ELI and HAp Materials

This section discusses the mechanical simulation results of the composite scaffold combined with HAp. This integrated structure represents the final construct of this study, as shown in Figure 7f and Table 6. Overall, the assembled model, including the titanium alloy scaffold, exhibits a maximum stress of 122.584 MPa. From the perspective of the brittle HAp material covering the central part, the maximum stress is only 27.92 MPa. It can be concluded that the maximum stress is concentrated on the titanium alloy scaffold, and the forced feedback on the bone contact surface due to this scaffold composition is subsequently discussed. In summary, the titanium alloy scaffold effectively protects the encapsulated HAp material during the initial stages of implantation, preventing compression-induced fractures. Additionally, the composition of the two materials with a radial gradient porosity design demonstrates minimal stress concentration, indicating a favorable mechanical response to the original bone tissue during the initial stages of implantation.

#### 3.1.6. Compilation and Discussion of Mechanical Simulation Data Results

The design featuring a radial gradient porosity of 123.7446 MPa is optimal regarding stress performance within the implanted structure. Adopting this approach and selecting HAp as an intermediary material for encapsulation results in a maximum stress of 122.8626 MPa on the Ti64 ELI scaffold and 27.92 MPa on the HAp. These outcomes closely resemble those achieved through autologous bone transplantation methods. Therefore, these comprehensive simulation results serve as a reference for subsequent processing, annealing procedures, and experimental testing of specimens.

### 3.2. Forming Conditions and Stress Relief Annealing

#### 3.2.1. Annealing Temperature and Time

**Figure 8:**
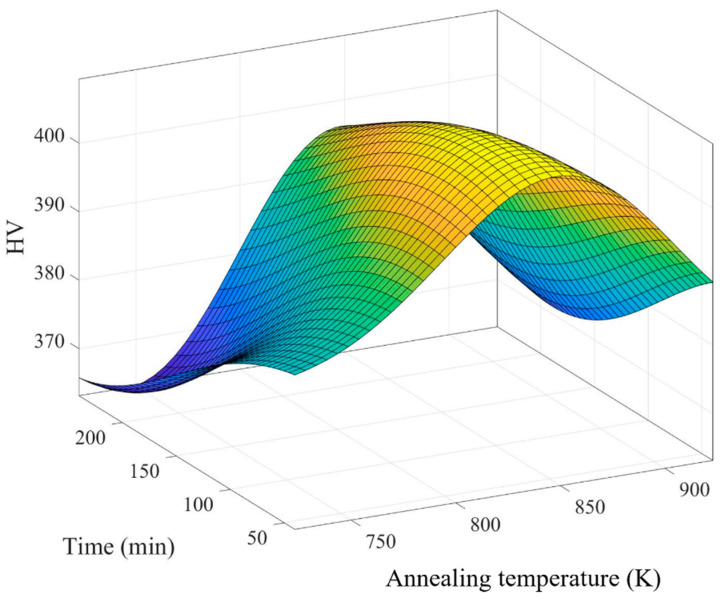
The optimal parameters for annealing.

Table 7 presents annealing data for the factors of time and temperature separated by the UDE. After annealing, the hardness of porous SLM Ti64 ELI was measured using an HMV-2 microhardness tester with a 4.903 N load for 15 s. The results indicate a significant change in hardness for the Ti64 ELI samples post-annealing. Hardness values for samples 1 to 6 ranged from 379.111 HV to 372.111 HV, surpassing the pre-annealing values of the original samples, which were approximately 340–350 HV, as shown in Table 8. Subsequently, the optimal annealing parameters were determined using MATLAB 2019, yielding a temperature of 842.8 K, a duration of 77.6 min, and an optimal hardness value of 409.425 HV, as depicted in Figure 8 and Table 9.

#### 3.2.2. SLM Process for the Fabrication of Scaffold Final Product

**Figure 9:**
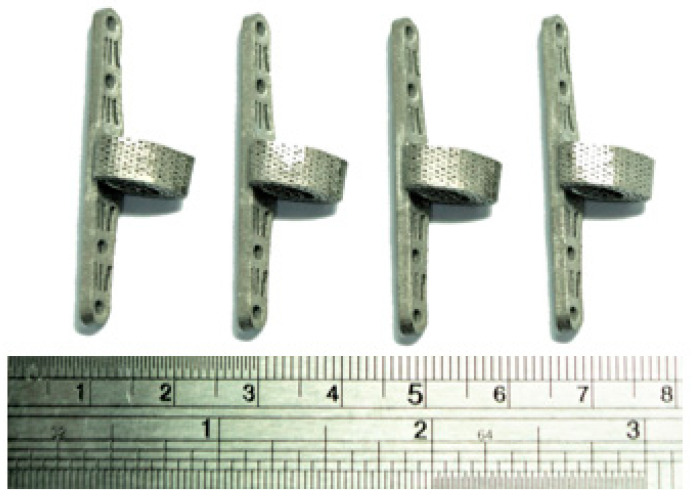
Titanium alloy specimen after completed processing.

This study utilized the SLM process to manufacture physical samples of the designed titanium alloy scaffold, as depicted in Figure 9. Upon visual inspection, the internal and external pores within the scaffold exhibited excellent formative outcomes. Before conducting mechanical property assessments and structural strength tests, a pre-processing step involving stress relief annealing was performed using a high-temperature furnace.

#### 3.2.3. Optimization of Stress Relief Annealing for Titanium Alloy Scaffold

**Figure 10:**
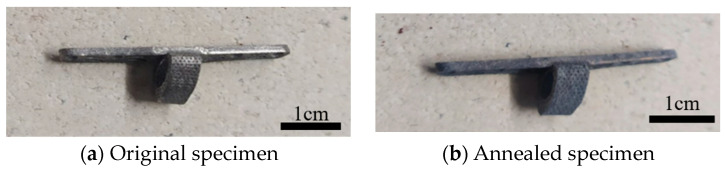
Specimens before and after stress relief annealing.

After the stress relief annealing treatment at high temperatures, the internal lattice structure of the melted titanium alloy specimens changes due to prolonged heating, releasing internal residual stresses. Concurrently, the material’s mechanical properties also undergo alterations, thereby enhancing the stability of the material structure, as illustrated in Figure 10. Subsequently, differences in the material’s mechanical properties were assessed using nanoindentation measurements.

### 3.3. Mechanical Properties and Strength Testing of Scaffold Samples

#### 3.3.1. Results of Material Mechanical Property Measurement Using Nanoindentation

Using a nanoindentation system, we compared the mechanical properties of the titanium alloy scaffold samples before and after annealing. The tested properties included the material’s Young’s modulus and hardness. Data analysis revealed that the original samples exhibited lower values in both parameters before annealing than the annealed samples. Furthermore, the standard deviation of the values measured at five points showed a decreasing trend after annealing. This indicates that removing residual stress enhanced the strength and stability of the titanium alloy material.

- Unannealed Sample Measurement

The unannealed samples were subjected to a five-point array test with a depth of 2000 nanometers using a nanoindentation tester. Load–displacement curves were plotted based on the values from the instrument’s internal sensors. The elastic modulus at each point was calculated using these curves and the relevant formulas. The elastic modulus was determined to be 124.31 ± 9.98 GPa and the hardness to be 3.91 ± 0.37 GPa (Table 10).

- Annealed Sample Measurement

The annealed specimens were tested using the same conditions used for the unannealed specimens. The elastic modulus was determined to be 130.58 ± 9.95 GPa and the hardness to be 4.11 ± 0.14 GPa (Table 11).

#### 3.3.2. Compression Test

This study utilized a universal testing machine to perform compressive strength tests on titanium alloy scaffold specimens. The specimens, divided into three groups for both annealed and unannealed conditions, underwent stress–strain curve analysis to assess overall structural strength. Strain energy calculations were employed to examine stress concentration tendencies. The analysis results indicate that the generated strain under identical loads is lower for pre- and post-annealed specimens, signifying a more stable overall structural behavior. The fitting of three stress–strain curves further validates this observation. The strain energy calculations demonstrate a notable reduction, indicating the efficacy of annealing in minimizing stress concentration and mitigating the stress shielding effect.

- Unannealed Sample Measurement

**Figure 11:**
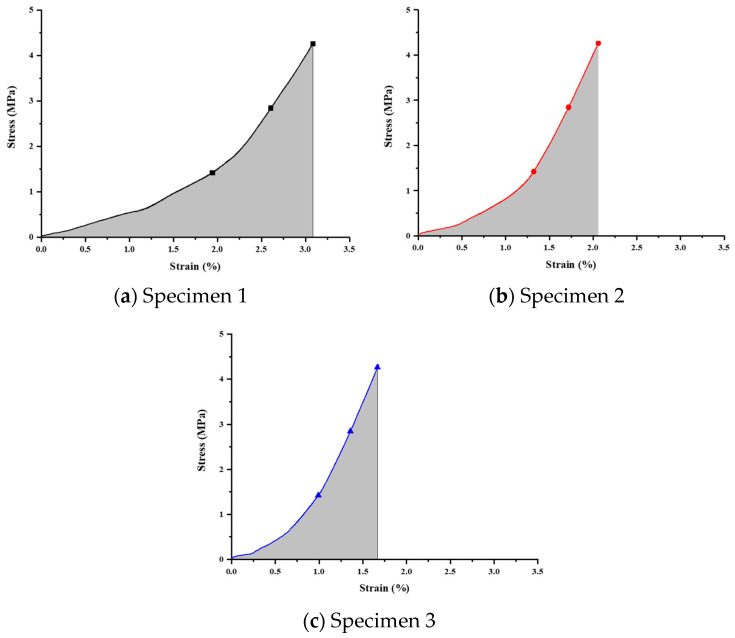
Stress–strain relationship curve for unannealed specimens.

**Figure 12:**
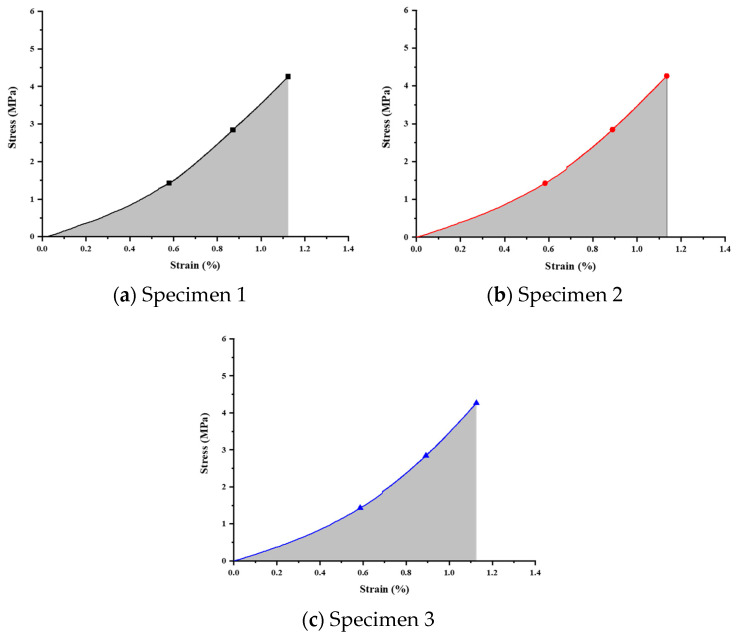
Stress–strain relationship curve for annealed specimens.

The compression test results (Figure 11) were analyzed, and stress-versus-strain curves were obtained (Figure 12 and Table 12). The curves represent the applied loads of 50 N, 100 N, and 150 N, with corresponding calculated strain energy values of 4.18755, 2.74358, and 2.38102 MPa, respectively.

- Annealed Sample Measurement

Compression tests on the annealed specimens were performed using identical measurement methods and boundary conditions. The results are presented in Figure 12 and Table 13, showing the corresponding calculated strain energies to be 1.85409, 1.87922, and 1.83082 MPa, respectively.

## 4. Conclusions

This study employed Ti64 ELI material to fabricate a composite scaffold structure featuring radial gradient porosity. A defect model in the tibia was established based on scanned images of a live rabbit’s skeletal structure. Finite element analysis using ANSYS-Workbench quantitatively evaluated the mechanical impact of various combinations on scaffold performance. The results indicated that the dual-material composite scaffold with a radial gradient porosity design replicated a stress distribution similar to autogenous bone grafts, demonstrating that the designed gradient porosity scaffold can effectively avoid the stress shielding effect. Subsequent experiments revealed that the stress relief annealing process reduced the residual stress in the scaffold. With annealing, both the elastic modulus and the hardness of the scaffold increased compared to the corresponding value for the unannealed scaffold. Compressive strength tests validated the enhanced structural strength of the annealed specimens. Under a load of 150 N, the scaffold’s strain energy was significantly reduced, which proves that this study’s design can effectively reduce the scaffold’s stress concentration. This study developed a radial gradient porosity scaffold with outstanding mechanical performance and structural stability. UDE dramatically simplifies the process of finding optimal annealing temperature parameters. SLM and stress relief annealing technologies effectively improved the material’s manufacturing efficiency and mechanical properties, holding practical significance for developing biocompatible and structurally stable scaffold materials. The study highlights the robust mechanical performance in the internal structure of titanium cages but identifies persistent stress concentration at structural connections. Future improvements should focus on optimizing the design by incorporating maximum roundness, a triple-curved surface, and increased thickness to address this. This aligns the design more closely with native bone tissue characteristics. The current study explores the bracket design on the tibia for fixing long limb bones. Future research should involve the proportional scaling of the titanium cage and its application to other limbs for a comprehensive comparative assessment of effects. After stress relief annealing, the surface oxidation of titanium alloy samples raises concerns about brittleness. Future investigations may explore alternative residual stress relief methods, such as ultra-low temperatures or vibrational stress relief, to maintain material flexibility while effectively relieving stress.

## Figures and Tables

**Table 1:** Finite element analysis parameters.

Scaffold Material Properties		Porosity (%)	Mesh Elements
Scaffold Outside	Scaffold Inside		
Cortical bone	Cancellous bone	-	110,297
Ti64 ELI	-	Gradient	912,873
Ti64 ELI	-	50%	2,219,942
Ti64 ELI	-	70%	1,368,419
Ti64 ELI	Ti64 ELI	Gradient	837,930
Ti64 ELI	HAp	Gradient	829,178

**Table 2:** A1.2 $U_{6}^{*}$(6^4^) table for six levels.

	1	2	3	4
1	1	2	3	6
2	2	4	6	5
3	3	6	2	4
4	4	1	5	3
5	5	3	1	2
6	6	5	4	1

**Table 3:** Using the form for $U_{6}^{*}$(6^4^) table.

S	Column Number				D
2	1	3			0.1875
3	1	2	3		0.2656
4	1	2	3	4	0.2990

**Table 4:** CSM machine parameter settings.

Parameter	Unit	Value
Depth Limit	nm	2000
Frequency Target	Hz	45
Surface Approach Velocity	nm/s	10
Minimum Calculation Depth	nm	500
Maximum Calculation Depth	nm	1500

**Table 5:** Boundary Conditions for Compression Test.

	Quantity	Mode	Load (N)	Testing Speed (kN/s)
Original specimen	3	Single-direction control	0–150	0.001
Annealed specimen				

**Table 6:** Stress and strain analysis results.

Specimens	Maximum Equivalent Stress (MPa)	Maximum Equivalent Strain (%)
Autologous bone graft block	14.7956	0.2514
50% porosity	170.7552	0.1588
70% porosity	538.8579	0.4983
Radial gradient porosity	123.7446	0.1146
Ti64 ELI support body	160.2153	0.1676
HAp gradient pore scaffolds	122.8626	0.1147

**Table 7:** The UDE method for annealing parameters.

No.	Annealing Temperature (K)	Annealing Time (min)
1	723	120
2	763	240
3	803	80
4	843	200
5	883	40
6	923	160

**Table 8:** Overall hardness for each specimen.

No.	HV (MPa)	σ (MPa)
1	379.111	2.183
2	365.111	2.055
3	401.778	3.794
4	400.667	4.546
5	402.889	3.247
6	372.111	2.331
Unannealed specimen	340~350	-

**Table 9:** Optimized values of the annealing temperature, annealing time, and hardness.

Annealing Temperature (K)	Annealing Time (min)	HV (MPa)
842.8	77.6	409.425

**Table 10:** Nanoindentation measurement results of unannealed samples.

Sample	Hardness (GPa)
Test 1	3.89
Test 2	3.36
Test 3	3.87
Test 4	4.00
Test 5	4.40
Average	3.91
Mean ± standard deviation	3.91 ± 0.37

**Table 11:** Nanoindentation measurement results of annealed specimens.

Sample	Hardness (GPa)
Test 1	4.25
Test 2	3.98
Test 3	4.10
Test 4	4.28
Test 5	3.97
Average	4.11
Mean ± standard deviation	4.11 ± 0.14

**Table 12:** Analysis of compression test results for unannealed specimens.

Specimen	Stress (MPa)/Strain			Mean Calculated Train Energy (MPa)
	50 N	100 N	150 N	
1	1.422/1.941	2.843/2.606	4.264/3.083	4.18755
2	1.421/1.321	2.843/1.720	4.264/2.060	2.74358
3	1.423/0.991	2.845/1.357	4.263/1.666	2.38102

**Table 13:** Analysis of compression test results for annealed specimens.

Specimen	Stress (MPa)/Strain			Mean Calculated Strain Energy (MPa)
	50 N	100 N	150 N	
1	1.424/0.580	2.843/0.871	4.265/1.124	1.85409
2	1.428/0.583	2.844/0.889	4.264/1.135	1.87922
3	1.429/0.587	2.843/0.892	4.264/1.126	1.83082

## Data Availability

The data presented in this study are available on request from the corresponding author.

## References

[1] Chen Z., Yan X., Yin S., Liu L., Liu X., Zhao G., Ma W., Qi W., Ren Z., Liao H. (2020). Influence of the pore size and porosity of selective laser melted Ti6Al4V ELI porous scaffold on cell proliferation, osteogenesis and bone ingrowth. Mater. Sci. Eng. C.

[2] Shum J.M., Gadomski B.C., Tredinnick S.J., Fok W., Fernandez J., Nelson B., Palmer R.H., McGilvray K.C., Hooper G.J., Puttlitz C. (2023). Enhanced bone formation in locally-optimized, low-stiffness additive manufactured titanium implants. An in silico and in vivo tibial advancement study. Acta Biomater..

[3] Alipour S., Nour S., Attari S.M., Mohajeri M., Kianersi S., Taromian F., Khalkhali M., Aninwene G.E., Tayebi L. (2022). A review on in vitro/in vivo response of additively manufactured Ti–6Al–4V alloy. J. Mater. Chem. B.

[4] Rojas A.R., Elguezabal A.A., Porporati A.A., Bernal M.B., Ponce H.E.E. (2023). Performance of Metals and Ceramics in Total Hip Arthroplasty.

[5] Zhou S.-J., Jiang W.-X., You J. (2018). Repair materials for bone defects: Present status, needs, and future developments. Chin. J. Tissue Eng. Res..

[6] Robinson P.G., Abrams G.D., Sherman S.L., Safran M.R., Murray I.R. (2020). Autologous bone grafting. Oper. Tech. Sports Med..

[7] Heimes D., Pabst A., Becker P., Hartmann A., Kloss F., Tunkel J., Smeets R., Kämmerer P.W. (2023). Comparison of morbidity-related parameters between autologous and allogeneic bone grafts for alveolar ridge augmentation from patients’ perspective—A questionnaire-based cohort study. Clin. Implant. Dent. Relat. Res..

[8] Li J., Wang Z., Guo Z., Chen G.J., Fu J., Pei G.X.J. (2010). The use of allograft shell with intramedullary vascularized fibula graft for intercalary reconstruction after diaphyseal resection for lower extremity bony malignancy. J. Surg. Oncol..

[9] Smith C.A., Richardson S.M., Eagle M.J., Rooney P., Board T., Hoyland J.A. (2015). The use of a novel bone allograft wash process to generate a biocompatible, mechanically stable and osteoinductive biological scaffold for use in bone tissue engineering. J. Tissue Eng. Regen. Med..

[10] Abbasi N., Hamlet S., Love R.M., Nguyen N.T. (2020). Porous scaffolds for bone regeneration. J. Sci. Adv. Mater. Devices.

[11] Alshammari A., Alabdah F., Wang W., Cooper G. (2023). Virtual Design of 3D-Printed Bone Tissue Engineered Scaffold Shape Using Mechanobiological Modeling. Relationship of Scaffold Pore Architecture to Bone Tissue Formation. Polymers.

[12] Hulbert S.F., Morrison S.J., Klawitter J.J. (1972). Tissue reaction to three ceramics of porous and non-porous structures. J. Biomed. Mater. Res..

[13] Taniguchi N., Fujibayashi S., Takemoto M., Sasaki K., Otsuki B., Nakamura T., Matsushita T., Kokubo T., Matsuda S. (2016). Effect of pore size on bone ingrowth into porous titanium implants fabricated by additive manufacturing: An in vivo experiment. Mater. Sci. Eng. C—Mater. Biol. Appl..

[14] Yu A.-H., Xu W., Lu X., Tamaddon M., Liu B.-W., Tian S.-W., Zhang C., Mughal M.A., Zhang J.-Z., Liu C.-Z. (2023). Development and characterizations of graded porous titanium scaffolds via selective laser melting for orthopedic applications. Trans. Nonferrous Met. Soc. China.

[15] Chang B., Song W., Han T., Yan J., Li F., Zhao L., Kou H., Zhang Y. (2016). Influence of pore size on porous titanium fabricated through vacuum diffusion bonding of titanium meshes on cell penetration and bone ingrowth. Acta Biomater..

[16] Oleksy M., Dynarowicz K., Aebisher D. (2023). Advances in Biodegradable Polymers and Biomaterials for Medical Applications—A Review. Molecules.

[17] Kutz M. (2003). Standard Handbook of Biomedical Engineering & Design.

[18] Arcos D., Vallet-Regi M. (2020). Substituted hydroxyapatite coatings of bone implants. J. Mater. Chem. B.

[19] Shah F.A., Snis A., Matic A., Thomsen P., Palmquist A. (2016). 3D printed Ti6Al4V implant surface promotes bone maturation and retains a higher density of less aged osteocytes at the bone-implant interface. Acta Biomater..

[20] Niinomi M., Boehlert C.J. (2015). Titanium alloys for biomedical applications. Advances in Metallic Biomaterials.

[21] Donachie M.J. (2000). Titanium: A Technical Guide.

[22] Luo J.P., Huang Y.J., Xu J.Y., Sun J.F., Dargusch M.S., Hou C.H., Ren L., Wang R.Z., Ebel T., Yan M. (2020). Additively manufactured biomedical Ti-Nb-Ta-Zr lattices with tunable Young’s modulus: Mechanical property, biocompatibility, and proteomics analysis. Mater. Sci. Eng. C.

[23] Song C., Chen J., Lei H., Yang Z., Deng Z., Li Y., Wang J., Yang Y., Han C. (2024). Radial gradient design facilitating the additive manufacturing of low-modulus gyroid tantalum structures. Int. J. Mech. Sci..

[24] Geetha M., Singh A.K., Asokamani R., Gogia A.K. (2009). Ti based biomaterials, the ultimate choice for orthopaedic implants—A review. Prog. Mater. Sci..

[25] Long M., Rack H. (1998). Titanium alloys in total joint replacement—A materials science perspective. Biomaterials.

[26] Zhang H.Q., Guo Q., Liu S.H., Guo C.F., Gao Q.L., Tang M.X. (2019). Comparison of mid-term outcomes of posterior or postero-anterior approach using different bone grafting in children with lumbar tuberculosis. Medicine.

[27] Hoffler C.E., Moore K.E., Kozloff K., Zysset P.K., Goldstein S.A. (2000). Age, gender, and bone lamellae elastic moduli. J. Orthop. Res..

[28] Rotta G., Seramak T., Zasińska K. (2015). Estimation of Young’s modulus of the porous titanium alloy with the use of FEM package. Adv. Mater. Sci..

[29] Chmielewska A., Dean D. (2023). The Role of Stiffness-Matching in Avoiding Stress Shielding-Induced Bone Loss and Stress Concentration-Induced Skeletal Reconstruction Device Failure. Acta Biomater..

[30] Li Z., Chen Z., Chen X., Zhao R. (2024). Multi-objective optimization for designing porous scaffolds with controllable mechanics and permeability: A case study on triply periodic minimal surface scaffolds. Compos. Struct..

[31] Wang Z., Liao B., Liu Y., Liao Y., Zhou Y., Li W. (2024). Investigating the impact of structural parameters in 3D-printed triply periodic minimal surface gyroid porous scaffolds on compression performance, cell response, and bone regeneration. J. Biomed. Mater. Res. Part B Appl. Biomater..

[32] Pan C.T., Lin C.H., Huang Y.K., Jang J.S., Lin H.K., Kuo C.N., Lin D.Y., Huang J.C. (2021). Design of customize interbody fusion cages of Ti64ELI with radial gradient porosity by selective laser melting process. Micromachines.

[33] Pan C.T., Hsu W.H., Cheng Y.S., Wen Z.H., Chen W.F. (2021). A New Design of Porosity Gradient Ti-6Al-4V Encapsulated Hydroxyapatite Dual Materials Composite Scaffold for Bone Defects. Micromachines.

[34] Gushue D.L., Houck J., Lerner A.L. (2005). Rabbit knee joint biomechanics: Motion analysis and modeling of forces during hopping. J. Orthop. Res..

[35] Perez M.A., Moreo P., Garcia-Aznar J.M., Doblare M. (2008). Computational simulation of dental implant osseointegration through resonance frequency analysis. J. Biomech..

